# Vaccines work

**DOI:** 10.1038/s41467-018-04085-z

**Published:** 2018-04-24

## Abstract

Vaccination has successfully reduced the burden of infectious diseases worldwide, but stagnating immunization coverage and lack of effective vaccines for many endemic and newly emerging pathogens pose a threat to sustainable global health. In light of World Immunization Week 2018, which highlights the importance of high vaccination coverage, *Nature Communications* is taking stock of current advances and barriers in vaccine development and distribution.

Vaccination is one of the most successful and cost-effective public health interventions. The World Health Organization (WHO) estimates that an average of 2 to 3 million deaths are prevented every year, thanks to worldwide vaccinations. Many more lives are protected from acute disease and life-long disabilities caused by infections.

“Increasing vaccination coverage and providing universal access to vaccines are crucial to realise sustainable global health and a fair chance of a healthy life for every child.”

The international community and policy-makers have long recognized the immense benefit of vaccination and have put a concerted effort and billions of dollars into global immunization campaigns; this has successfully reduced burden of infectious diseases. For example, in 1988, at the beginning of the Global Polio Eradication Initiative (GPEI), ~1000 children worldwide were paralyzed every day due to poliomyelitis. Today, most countries are free of polio and only three countries—Afghanistan, Nigeria, and Pakistan—remain endemic. A polio-free world is within reach.

Yet polio is only one of currently 25 vaccine-preventable infections^1^. Global immunization coverage is too low and an estimated 1.5 million deaths could be avoided by “simply” increasing the number of vaccinated children^2^.

One reason for stagnating or declining vaccination coverage in many regions of the world is a lack of resources. The Global Alliance for Vaccines and Immunizations (Gavi) was created in 2000 to address this problem and increase access to vaccines in lower-income countries. Gavi has contributed to vaccination of roughly 640 million children. It aims to strengthen health systems and improve sustainability of national immunization programs, with the ultimate goal of enabling developing countries to carry out vaccination independently. This approach has shown success and several of the initially included countries no longer depend on financial support from Gavi.

Another barrier is a societal resistance to vaccination, largely rooted in an unfounded mistrust and unreasonable fear from adverse side effects. Measles is a prime example. A safe vaccine has been available since the 1960s and has protected millions of children’s lives worldwide. But vaccination coverage is currently too low to prevent outbreaks. In 2017, 14,451 measles cases were reported in Europe, a more than three-fold increase from 2016^3^. Measles infection routinely causes complications and approximately one of every 1000 measles infected children will die. In contrast to common misperception, this holds true in developed countries with state of the art healthcare. Italy for example reported roughly 4400 measles cases between January and August of 2017, nearly 2000 of which were hospitalized—three children died^4^.

This year’s World Immunization Week with the theme “Protected Together, #VaccinesWork” highlights the importance of immunization and encourages everyone, from donors to the general public, to help to increase vaccination coverage. We can all do our part by getting vaccinated.

Reaching high coverage for already approved vaccines is not the only challenge we are facing. We currently lack vaccines for many infectious diseases, including respiratory and diarrheal diseases, fungi, HIV, tuberculosis, malaria, and emerging pathogens. Vaccine development for many of these diseases has proven a formidable challenge, and previously successful approaches are unlikely to be sufficient.

There is cause for optimism. Technological advances have opened up promising new avenues in vaccine research and development, including antigen discovery and design. Antibodies isolated from protected individuals pinpoint to immunogenic areas in microbial molecules^5^. Structure-based antigen design stabilizes and exposes epitopes to guide the immune response to highly conserved domains^6^. Nanoparticles enhance stability and modify immunogenicity of selected molecules^7^. mRNA vaccines serve as a new platform in clinical development that, in addition to other advantages, promise rapid, and inexpensive production^8^. This non-exhaustive list illustrates encouraging progress in design of next generation vaccines.

Rational antigen design and vaccine development crucially rely on an in depth understanding of the protective immune response. Yet animal models are often poor predictors of the human immune response to a particular pathogen, rendering specimens from naturally infected individuals invaluable. Each sample may be interrogated with a multitude of assays, including serology, transcriptomics, metabolomics, and others. Not surprisingly, systems vaccinology plays an increasingly important role to make sense of the wealth of data^9^. Identified correlates of protection may be used as a surrogate of vaccine efficacy during development and enable objective comparison of vaccine candidates.

Natural infection is not the only option to study the human immune response. Deliberate infection of volunteers under controlled conditions, so-called human challenge studies, represent an intriguing alternative, at least for some pathogens. While such experiments may have a checkered history, regulations, and ethical guidelines are in place today to ensure safety and proper utilization^10^.

Despite advances in research and development, vaccines for many emerging pathogens are unlikely to be developed, largely due to uncertainties in regulatory pathways and markets. This leaves us vulnerable to large-scale outbreaks of emerging viruses, such as Ebola and Zika, with devastating effects on health and tremendous socioeconomic costs. To address this, the Coalition for Epidemic Preparedness Innovations (CEPI) was founded in 2016. CEPI facilitates and finances vaccine development for high risk pathogens to the stage of a safe and immunogenic candidate, with the goal to enable rapid effectiveness trials in case of an epidemic.

There are already encouraging success stories of similar initiatives. The Meningitis Vaccine Project (MVP) was launched in 2001 to develop a vaccine protecting from yearly meningitis outbreaks in sub-Saharan Africa. It was clear that the vaccine would need to cost less than 50 US cents per dose to be affordable, which is about one-tenth of a typical vaccine. MVP succeeded with a hitherto unconventional approach—they developed the vaccine together with a manufacturer in India. This is the first vaccine specifically developed for Africa.

Vaccines are arguably the best approach to stop infectious diseases, and benefits can go beyond individual and public health gains^11^. Non-health outcomes include, for example, higher educational attainment^12^, which may affect a child’s economic future. Increasing vaccination coverage and providing universal access to vaccines are crucial to realise sustainable global health and a fair chance of a healthy life for every child.
